# Supplementary material for: Maternal Exposure to Ambient Air Pollution and Pregnancy Complications in Victoria, Australia
Source: Int J Environ Res Public Health. 2020 Apr 9;17(7):2572. doi: 10.3390/ijerph17072572 (PMC7178226; doi:10.3390/ijerph17072572)
Supplement: Supplementary file 1 [file ijerph-17-02572-s001.pdf]

Supplementary Material

Table S1. List and coordinates of Bureau of Meteorology weather stations used to determine ambient temperature.

Weather station name	Coordinates (Latitude, longitude °C)
Ararat Prison	37.27, 142.98
Bairnsdale Airport	37.88, 147.57
Ballarat Aerodome	37.51, 143.79
Benalla Airport	36.55, 145.99
Bendigo Airport	36.74, 144.33
Breakwater (Geelong Racecourse)	38.17, 144.38
Bundoora	37.72, 145.04
Castlemaine Prison	37.08, 144.24
Coldstream	37.72, 145.41
Cranbourne Botanic Gardens	38.13, 145.26
East Sale	38.11, 147.13
Essendon Airport	37.73, 144.91
Frankston Aws	38.15, 145.12
Hamilton Airport	37.65, 142.06
Laverton RAAF	37.86, 144.76
Maryborough	37.06, 143.73
Melbourne Airport	37.67, 144.83
Mildura Airport	34.24, 142.09
Moorabbin Airport	37.98, 145.09
Mortlake racecourse	38.07, 142.77
Morwell	38.21, 146.47
Rutherglen Research	36.10, 146.51
Scoresby Research Institute	37.87, 145.26
Sheoaks	37.91, 144.13
Shepparton Airport	36.43, 145.39
Swan Hill Aerodome	35.38, 143.54
Tatura Inst Sustainable Ag	36.44, 145.27
Viewbank	37.74, 145.09
Warrnambool Airport	38.29, 142.43
Wonthaggi	38.61, 145.59
Yarrawonga	36.03, 146.03

Table S2. Ambient NO_2 and $PM_{2.5}$ in Greater Melbourne compared with the rest of Victoria for births occurring in Victoria between 1 March 2012 and 31 Dec 2015.

	Greater Melbourne			Rest of Victoria		
	Mean	Median	Q25, Q75	Mean	Median	Q25, Q75
**Ambient NO_2 concentration (ppb)**	6.6	6.3	4.6, 8.2	3.6	3.2	2.5, 4.1
**Ambient $PM_{2.5}$ concentration (μg/m³)**	7.0	7.1	6.5, 7.7	6.9	7.1	6.0, 7.9

Table S3. Association between average ambient NO_2 and $PM_{2.5}$ exposure in pregnancy and selected pregnancy conditions using single year exposure assignment (year of conception only) for births in Victoria, Australia between 1 March 2012 and 31 Dec 2015.

	Per IQR increase in annual ambient NO_2 concentration (ppb)	Per IQR increase in annual ambient PM2.5 concentration (μg/m³)
**Pregnancy condition**	**Relative Risk (95%CI); p value**	

Hypertensive disorder of pregnancy (pregnancy-induced hypertension, preeclampsia and eclampsia)*	**0.97 (0.96, 0.97); <0.0001**	**0.96 (0.95, 0.98); <0.0001**
**Gestational Diabetes Mellitus****	**0.98 (0.98, 0.99); <0.0001**	1.01 (0.99, 1.02): 0.10
**Placental abruption****	**0.95 (0.91, 0.99); 0.01**	1.04 (0.95, 1.14); 0.42

**Adjusted for maternal age >=35, parity, Index of Relative Socioeconomic Disadvantage, average ambient temperature whole of pregnancy*

*** Adjusted for smoking in early pregnancy, maternal age (years), parity, Index of Relative Socioeconomic Disadvantage, year of conception, average ambient temperature whole of pregnancy*

Bold typeface indicates statistically significant p<0.05

Table S4. Association between average ambient NO_2 and $PM_{2.5}$ exposure in pregnancy and selected pregnancy conditions using single pollutant models for births in Victoria, Australia between 1 March 2012 and 31 Dec 2015.

	**Per IQR increase in ambient NO_2 concentration (ppb)**	**Per IQR increase in ambient $PM_{2.5}$ concentration (µg/m³)**
**Pregnancy condition**	**Relative Risk (95%CI); p value**	
**Hypertensive disorder of pregnancy (pregnancy-induced hypertension, preeclampsia, eclampsia)***	**0.86 (0.84, 0.88); <0.0001**	**0.92 (0.90, 0.94); <0.0001**
**Gestational Diabetes Mellitus****	**0.92 (0.92, 0.95); <0.0001**	0.99 (0.98, 1.01); 0.20
**Placental abruption****	**0.82 (0.71, 0.96); 0.01**	0.99 (0.89, 1.11); 0.90

**Adjusted for maternal age >=35, parity, Index of Relative Socioeconomic Disadvantage, average ambient temperature whole of pregnancy*

*** Adjusted for smoking in early pregnancy, maternal age (years), parity, Index of Relative Socioeconomic Disadvantage, year of conception, average ambient temperature whole of pregnancy*

Bold typeface indicates statistically significant p<0.05

Table S5. Association between average ambient NO_2 and $PM_{2.5}$ exposure in pregnancy and selected pregnancy conditions adjusting for remoteness for births in Victoria, Australia between 1 March 2012 and 31 Dec 2015.

	**Per IQR increase in ambient NO_2 concentration (ppb)**	**Per IQR increase in ambient $PM_{2.5}$ concentration (µg/m³)**
**Pregnancy condition**	**Relative Risk (95%CI); p value**	
**Hypertensive disorder of pregnancy (pregnancy-induced hypertension, preeclampsia, eclampsia)***	**0.92 (0.89, 0.95); <0.0001**	**0.94 (0.92, 0.97); <0.001**
**Gestational Diabetes Mellitus****	**0.93 (0.91, 0.95); <0.0001**	**1.02 (1.00, 1.03); 0.04**
**Placental abruption****	**0.80 (0.66, 0.97); 0.02**	1.07 (0.94, 1.21); 0.32

**Adjusted for maternal age >=35, parity, Index of Relative Socioeconomic Disadvantage, average ambient temperature whole of pregnancy, remoteness*

*** Adjusted for smoking in early pregnancy, maternal age (years), parity, Index of Relative Socioeconomic Disadvantage, year of conception, average ambient temperature whole of pregnancy, remoteness*

Bold typeface indicates statistically significant $p<0.05$

Table S6. Association between average ambient NO_2 and $PM_{2.5}$ exposure in pregnancy and selected pregnancy conditions among term births only (>=37 and <=42 weeks gestation) in Victoria, Australia between 1 March 2012 and 31 Dec 2015.

	**Per IQR increase in annual ambient NO_2 concentration (ppb)**	**Per IQR increase in annual ambient $PM_{2.5}$ concentration ($\mu g/m^3$)**
**Pregnancy condition**	**Relative Risk (95%CI); p value**	
**Hypertensive disorder of pregnancy (pregnancy-induced hypertension, preeclampsia, eclampsia)***	**0.88 (0.85, 0.91); <0.0001**	**0.93 (0.91, 0.95); <0.0001**
**Gestational Diabetes Mellitus****	**0.92 (0.90, 0.94); <0.0001**	1.02 (1.00, 1.03); 0.05
**Placental abruption****	**0.76 (0.59, 0.97); 0.03**	1.15 (0.95, 1.40); 0.15

**Adjusted for maternal age >=35, parity, Index of Relative Socioeconomic Disadvantage, average ambient temperature whole of pregnancy*

*** Adjusted for smoking in early pregnancy, maternal age (years), parity, Index of Relative Socioeconomic Disadvantage, year of conception, average ambient temperature whole of pregnancy*

Bold typeface indicates statistically significant $p<0.05$

Table S7. Association between average ambient NO_2 and $PM_{2.5}$ exposure in pregnancy and selected pregnancy conditions adjusting for fixed cohort bias (n = 257, 173).

	**Per IQR increase in annual ambient NO_2 concentration (ppb)**	**Per IQR increase in annual ambient $PM_{2.5}$ concentration ($\mu g/m^3$)**
**Pregnancy condition**	**Relative Risk (95%CI); p value**	
**Hypertensive disorder of pregnancy (pregnancy-induced hypertension, preeclampsia, eclampsia)***	**0.92 (0.88, 0.95); <0.0001**	**0.94 (0.92, 0.96); <0.0001**
**Gestational Diabetes Mellitus****	**0.93 (0.91, 0.95); <0.0001**	**1.02 (1.00, 1.03); 0.05**
**Placental abruption****	**0.81 (0.66, 0.98); 0.034**	1.07 (0.94, 1.22); 0.28

**Adjusted for maternal age >=35, parity, Index of Relative Socioeconomic Disadvantage, average ambient temperature whole of pregnancy*

*** Adjusted for smoking in early pregnancy, maternal age (years), parity, Index of Relative Socioeconomic Disadvantage, year of conception, average ambient temperature whole of pregnancy*

Footnote: includes pregnancies with estimated conception date <22 weeks before cohort start date and >42 weeks before the cohort end date (estimated date of conception 29/09/2011 to 12/03/2015)

Bold typeface indicates statistically significant $p<0.05$
